# Mediterranean-Oriented Dietary Intervention Is Effective to Reduce Liver Steatosis in Patients with Nonalcoholic Fatty Liver Disease: Results from an Italian Clinical Trial

**DOI:** 10.1155/2024/8861126

**Published:** 2024-01-25

**Authors:** Barbara Zanini, Federica Benini, Monica Marullo, Anna Simonetto, Angelo Rossi, Paola Cavagnoli, Alessia Bonalumi, Silvia Marconi, Marie Graciella Pigozzi, Gianni Gilioli, Alessandra Valerio, Francesco Donato, Maurizio Castellano, Chiara Ricci

**Affiliations:** ^1^Department of Clinical and Experimental Sciences, University of Brescia, Viale Europa 11, Brescia, Italy; ^2^Department of Medicine, ASST Spedali Civili of Brescia, Piazzale Spedali Civili 1, Brescia, Italy; ^3^Department of Medical and Surgical Specialties, Radiological Sciences, and Public Health, University of Brescia, Viale Europa 11, Brescia, Italy; ^4^Department of Civil Engineering, Architecture, Land and Environment, and Mathematics, University of Brescia, Viale Europa 11, Brescia, Italy; ^5^Health Protection Agency, ATS Brescia, Lombardy, Italy; ^6^Department of Molecular and Translational Medicine, University of Brescia, Viale Europa 11, Brescia, Italy

## Abstract

**Results:**

One hundred and fifty five subjects aged 20–59 years underwent (i) liver ultrasound (US), (ii) clinical and anthropometric evaluations, (iii) blood tests, and (iv) assessment of dietary habits. According to US evaluation, 73 of them had severe, moderate, or mild liver steatosis (NAFLD patients) and 82 had no liver steatosis (healthy controls). Fifty-eight NAFLD patients and 73 controls completed the study. Among NAFLD patients, 26 (45%) downgraded steatosis severity, 12 of which achieved complete steatosis regression (21%). Three of the healthy controls developed NAFLD. The NAFLD patients improved their dietary habits and reduced BMI and waist circumference, during the study period, more than healthy controls. Liver steatosis remission/regression was independent of changes in BMI or liver enzymes and was more frequent among patients with mild steatosis at baseline.

**Conclusions:**

Mediterranean dietary advices, without a personalised meal planning, were efficient in reducing/remitting NAFLD, especially among patients with mild disease, which argues in favour of early identification and lifestyle intervention. This trial is registered with NCT03300661.

## 1. Introduction

Nonalcoholic fatty liver disease (NAFLD) is a common liver disorder, characterized by excessive intrahepatic fat accumulation and by a broad spectrum of liver conditions, ranging from simple steatosis to steatohepatitis and cirrhosis [1].

NAFLD is often associated with systemic metabolic dysfunction. Recently, a panel of international experts from 22 countries proposed the new acronym MAFLD (metabolic dysfunction-associated fatty liver disease) to give relevance to the frequent association with type II diabetes, overweight/obesity, and other evidence of metabolic dysregulation [2]. Even if a consensus about nomenclature is not still reached, fatty liver disease (FLD) is an increasing worldwide condition, and the risk of disease progression and decompensation represents a leading cause of liver-related morbidity and mortality [3]. With an estimated European prevalence of 24%, ranging from 5 to 44% in different countries, FLD represents an important challenge in clinical practice in terms of screening, diagnosis, and identification of patients at risk of progression and with urgent need of management [4].

In the diagnostic algorithm of NAFLD, it is crucial to identify steatosis in the absence of a secondary cause, such as heavy alcohol consumption or steatogenic drug adoption [5]. In clinical practice, liver ultrasound is the most widespread diagnostic tool, given its accessibility and low cost, despite some limitations due to interoperator variability and poor sensitivity in detecting <20% liver fat [5]. Once steatosis is diagnosed, the major challenge for clinicians is to identify patients with NAFLD at the highest risk for liver-related or metabolic complications, in order to propose adequate management strategies, aimed at stopping disease progression [6, 7]. According to NICE guidelines, up-to-date lifestyle modifications on diet and physical activity, supported by a multidisciplinary team, represent the only evidence-based management for people with NAFLD [8]. All patients should be treated with the recommendations on physical activity and diet according to “NICE's obesity and preventing excess weight gain” guidelines, considering that a healthy diet and adequate physical activity may reduce liver fat content independently of weight reduction [8].

According to a recent review with a meta-analysis of 8 randomized clinical trials investigating the effect of different dietary interventions without exercise advice on patients with NAFLD, the Mediterranean diet, even without caloric restriction, was helpful in reducing liver fat, but diet without exercise did not lead to significant changes in liver enzymes, lipid profile, fasting glucose, or insulin [9]. Regarding physical activity intervention, at present there is still no agreement on the type, duration, and intensity of physical activity that would bring the best results for patients with NAFLD [10].

In order to assess the efficacy and feasibility of a Mediterranean-oriented lifestyle intervention to reduce liver steatosis in NAFLD patients, we performed a semipersonalized dietary counselling, implemented and supervised by a multidisciplinary team. For comparison, we also enrolled a group of healthy subjects without liver steatosis at baseline, who underwent the same evaluations as the NAFLD patients but did not receive the semipersonalized lifestyle intervention.

## 2. Methods

The nonrandomized clinical trial SEELN (from the Italian “Steatosi Epatica nonalcolica: Epidemiologia nutrizionale and Lifestyle mediciNe”) was approved by the Ethics Committee of Brescia District (assigned code NP2587), on January 2017, and was registered on clinicaltrial.gov with identifier number NCT03300661. The investigators designed the study in 2016 and considered as reference the last available update of NAFLD practice guidelines by the American Association for the Study of the Liver Diseases, published in 2012 [11].

First, a network of collaborating general practitioners (GPs) living in Brescia District was established. Second, the GPs invited their patients without a diagnosis of NAFLD to participate in the study, according to the selection criteria: age between 20 and 59 years, absence of a severe clinical condition, exclusion of pregnancy or breastfeeding, and absence of liver disease and related risk factors (alcohol heavy intake, hepatitis virus infection, hemochromatosis, and others).

Third, the selected subjects were admitted to the Department of Medicine, ASST Spedali Civili of Brescia, to undergo, after signing informed consent: (i) liver ultrasound (US), (ii) clinical and anthropometric evaluations, (iii) blood tests, and (iv) dietary habits assessment. Performed blood tests included cholesterol (total and divided into high density lipoprotein and low density lipoprotein), triglycerides, transaminases, fasting glycemia, and insulin level. Dietary habits were collected by a registered dietician and assessed using a self-administered Italian semiquantitative food frequency questionnaire (FFQ), and the development and validation process of the FFQ were part of the study protocol [12].

We developed a Mediterranean diet adherence score (Medscore) ranging from 0 to 25 (the higher the score, the better the adherence), based on a specific set of food items from the FFQ. Despite the availability of other validated scores to assess adherence to Mediterranean diet (most from Spain and Greece), we decided to create a new score specifically targeted to Italian population and with more food items, specifically chosen among the 146 foods of our FFQ. Details on Medscore assignment algorithm are provided in Table S1 (Supplementary Materials). We assessed the correlation between our new Medscore and the validated 14-item PREDIMED questionnaire score among 58 patients and 72 healthy subjects finding the following result: *r* = 0.3, *P* < 0.0001 [13].

According to the US evaluation, the subjects were classified into NAFLD patients, with severe, moderate, or mild steatosis or healthy controls, without steatosis. The US evaluation was performed always by the same two investigators (FB and CR), who decided together about steatosis presence and its degree.

The NAFLD patients, regardless of steatosis grade, underwent a clinical evaluation every 3 months for a total of a 12-month intervention. During clinical evaluation, weight, height, and waist circumference were assessed, and FFQ was administered and evaluated in order to increase and decrease frequency consumption of healthy and unhealthy foods, respectively. The proposed food consumption frequencies were based on the Mediterranean model and the official Mediterranean pyramid infographic was used during the nutrition education interview [14]. A 16-page booklet was delivered to each patient, in order to track the dietary suggestions and the anthropometric measures every 3 months (supplementary file 1, for review only). The booklet emphasized the importance of home cooking and promoted the regular consumption of whole grains, legumes, fish, olive oil, nuts, fruits, and vegetables and the reduction in the consumption of ultraprocessed foods, red meats, and processed meats. References to standard proportion of food categories, according to LARN (the Italian nutritional guidelines about recommended level of assumption of nutrients) was also included [15]. To enhance patients' adherence to dietary advice and to encourage home cooking, the dietician delivered, at any time point, three simple and tasty culinary recipes of typical Mediterranean dishes; no calorie counting and restriction neither personalised meal planning were provided, and for this reason, we considered our dietary intervention “semipersonalised.”

The control group received written general advice based on current Italian food-based dietary guidelines and did not undergo physical examinations every three months (supplementary File 2, for review only).

After 12 months, both NAFLD patients and healthy controls again underwent liver US, clinical and anthropometric evaluations, and repeated blood tests and filled in the FFQ. This second US evaluation was performed by the same investigators as the first one (FB and CR) and, while performing this second liver examination, they were blinded to the result of the first assessment. Descriptive statistics were reported for all variables, particularly arithmetic means with SD and proportions for continuous and categorical variables, respectively. A comparison between the NAFLD patients and healthy controls for each variable at baseline was performed using the nonparametric Mann–Whitney test, due to nonnormal distribution of all variables. The mean difference and SD between the baseline and 12-month values for each variable were computed for both NAFLD patients and healthy controls separately and tested using the Wilcoxon nonparametric test for paired data. Finally, a comparison of the mean differences between the baseline and 12-month values for each variable was performed between NAFLD patients and healthy controls using the Mann–Whitney test. All the statistical tests were two-sided with a 5% threshold for rejecting the null hypothesis, using the Stata program for PC, 14.0 version.

The sample size was based on the expected result of a partial or overall regression of liver steatosis in at least 50% of NAFLD patients, in line with the results of other dietary interventions [9]. Assuming a 95% confidence interval (CI) of this proportion of ±15% (from 35% to 65%), 50 subjects would have been enough. However, we decided to recruit at least 70 NAFLD patients for possible loss during the study, due to the relatively long duration of the intervention (12 months).

## 3. Results

**Figure 1:**
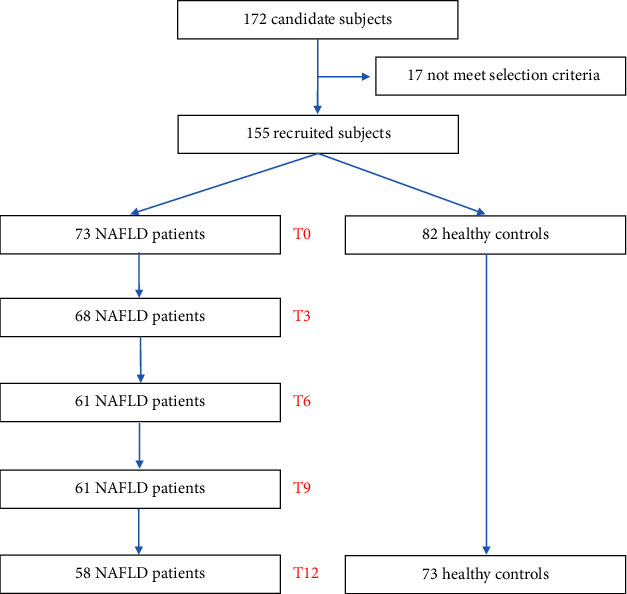
Flowchart of the study. T0: time 0 (baseline); T3: 3 months; T6: 6 months; T9: 9 months; T12: 12 months.

From June 2017 to April 2018, 172 candidate subjects were admitted for a preliminary evaluation. Seventeen subjects did not meet the selection criteria and were excluded. One hundred and fifty five subjects signed the informed consent, underwent liver ultrasound, and were assigned to the NAFLD (73 patients) or to the healthy control group (82 healthy subjects), according to the presence or absence of liver steatosis, respectively (Figure 1). The number of participating NAFLD patients reduced at each 3-month evaluation. Overall, 58 patients and 73 controls completed the study, with a drop-out rate of 20.5% and 11.0%, respectively.

The characteristics of the NAFLD patients and healthy subjects at baseline are reported in Table 1. As expected, BMI and waist circumference were significantly higher in the NAFLD than in the control group, whereas no differences were found as regards the other characteristics between the two groups, apart from education, which was on average higher among healthy subjects than among NAFLD patients (Table 1).

All the laboratory parameters indicative of risk of cardiovascular, metabolic, or liver disease showed a different distribution between the two groups: the NAFLD patients showed a worse pattern of laboratory parameters and Medscore than the healthy controls (Table 2).

**Figure 2:**
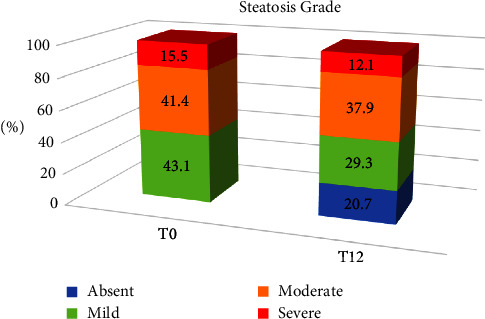
Steatosis grade of NAFLD patients at baseline and after 12 months of lifestyle intervention.

At baseline, 25 NAFLD patients (43.1%) presented mild, 24 NAFLD patients (41.4%) presented moderate, and 9 NAFLD patients (15.5%) presented severe steatosis. After 12 months of dietary and physical activity intervention, 26 NAFLD patients (44.8%) showed a regression of steatosis, and among them, 12 (20.7%) had a complete remission.  Figure 2 reports the steatosis grade at baseline (T0) and after 12 months of intervention (T12), according to liver US evaluation. Healthy control group did not present steatosis at baseline, but 3 of them developed liver steatosis, as found at the 12-month evaluation: 2 subjects developed mild and 1 moderate grade steatosis (data not shown in figure).

A complete remission of steatosis was observed in 9 of the 25 subjects with mild (36%) and 3 of the 33 ones (9%) with moderate or severe steatosis at baseline (*P*=0.02 by exact test).

Among the NAFLD patients, the 15 enrolled subjects who did not complete the study were not different from the others as regards the characteristics at baseline reported in Tables 1 and 2 (data not shown in table). Nine of them had mild and 6 moderate steatosis. Accordingly, the 9 healthy controls who did not undergo the 12-month final evaluation were not different from those who did (data not shown in table). As shown in Table 3, some parameters improved significantly among NAFLD patients from before to after intervention: BMI reduced, HDL cholesterol increased, and triglycerides decreased, on the average. The Medscore increased significantly in the NAFLD group. No change was observed in the control group in the parameters investigated and in Medscore from the baseline to 12-month evaluation. Considering the NAFLD patients with or without a BMI decrease of at least one point, from baseline to 12th month, 12 of the 23 (52%) subjects with BMI decline and 14 of the 35 (40%) ones with less or no BMI decline had steatosis regression (*P*=0.3) (data not shown in table).

Similar changes of the investigated parameters were observed from baseline to 12th month in both the 26 patients who achieved regression of steatosis and in the 32 ones with no regression or worsening of steatosis, without statistically significant differences between them (Table 4).

**Figure 3:**
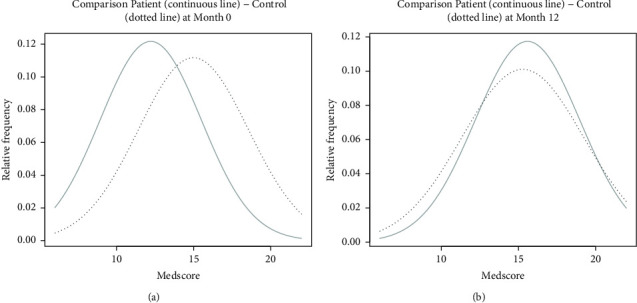
Plot of Medscore distribution among NAFLD patients (continuous line) and controls (dotted line) at baseline (T0) (a) and at 12th month (T12) (b).

The Medscore distribution curves in NAFLD patients and healthy subjects at baseline and at the end of the study are plotted in Figures 3(a) and 3(b), respectively. The Medscore curves are substantially different in the two groups at baseline (T0), with NAFLD patients showing lower values than healthy controls but are similar after 12 months, and the NAFLD patients having achieved Medscores similar to those observed in healthy controls (Figure 3(b)).

## 4. Discussion

Our Mediterranean-oriented intervention study in patients with NAFLD focused primarily on finding a strategy that was both effective in healing the disease and easy to implement in clinical practice. Lifestyle change is the first-line therapy for patients with NAFLD, but this is one of the most difficult challenges in healthcare settings [16, 17]. Our study showed a liver steatosis regression in about half of the NAFLD patients, almost all overweight or obese, participating in a 12-month lifestyle intervention. Using a score of adherence to a Mediterranean diet, we found that the NAFLD patients modified significantly their dietary habits from baseline to the end of the intervention. For comparison, a control group of healthy subjects without steatosis at baseline, who received only generic advice on healthy lifestyle, did not modify their habits, in spite of having undergone physical, instrumental, and laboratory evaluations, and some of them developed liver steatosis in the 12-month study time.

In order to improve the feasibility of a lifestyle change program, our intervention was only partially personalized: we first collected information on each patient's diet to better suggest a few, simple and acceptable, modifications of his/her dietary habits, but we did not provide personalized meal planning. We did not calculate the total daily energy expenditure for each patient, because we focused our intervention on diet quality rather than on calorie restriction. As suggested by other authors, the Mediterranean diet has the potential to improve fatty liver, even without reducing body weight, and it is recommended as the diet of choice for the management of NAFLD at present [1, 18, 19]. Indeed, although we observed a mean BMI decrease from baseline to the end of the intervention in NAFLD subjects, the liver steatosis regression was independent of BMI decline, as we found a similar proportion of steatosis regression in patients with and in those without a one-point BMI decrease. Of note, the liver steatosis regression was also independent of changes in common parameters of cardiovascular and metabolic risks such as glycemia, insulin resistance, total and LDL cholesterol levels.

Similar to previous studies, the steatosis improvement following better adherence to Mediterranean diet was also independent of AST and/or ALT decrease [20, 21]. As discussed in a recent review by Zelber-Sagi et al., the plausible biological mechanisms for this beneficial effect include the reduction of insulin resistance [18], although in our study, we found no change in glycemia, insulin level, and HOMA-IR among NAFLD patients, during the intervention period. As expected, NAFLD patients had a lower mean Medscore than the healthy controls at baseline [22]. The intervention was successful in improving adherence to the Mediterranean diet in the former, as shown by the distribution of Medscore values in the two groups. Indeed, the Medscore distribution curves among NAFLD patients and healthy subjects were different at the beginning of the study but overlapped at the end of the intervention, with a statistically significant improvement only among NAFLD patients (Table 4 and Figure 3).

In our study, regression in steatosis was achieved in 45% of cases, with complete remission (absence of steatosis according to liver US) in 21% of patients, after 12 months of lifestyle intervention. These success rates are quite in line with similar studies [19]. In particular, our results are similar to the single-arm intervention trial by Vilar-Gomez et al.: in 293 patients with nonalcoholic steatohepatitis (NASH), who underwent lifestyle changes for 52 weeks, a reduction and a resolution of steatohepatitis were achieved in 47% and 25% of the patients, respectively [23]. Contrary to Vilar-Gomez et al.'s study, however, we did not observe a higher weight loss among patients with improvement in steatosis than those without improvement (Table 4). The majority of patients who achieved complete remission of steatosis had mild steatosis at baseline. This finding underlines the importance of early identification of NAFLD in primary care in order to achieve the reversion of the disease [24–26].

Our study has various strengths. The SEELN project involved GPs in both design and active enrollment of the patients: the involvement of GPs is usually considered crucial for the early detection of NAFLD and for enhancing the patients' motivation before addressing them to specialists. The team of doctors and dieticians in the Department of Medicine was multidisciplinary and remained the same throughout the study. The methods for assessing dietary habits were standardized and validated [12, 14]. A registered dietician provided a written counselling to each patient on how to improve his/her diet at any time point, after a quick analysis of food consumption frequencies over the last three months and reinforced the advice with simple, tasty, and healthy cooking recipes. Two key elements of our intervention are the quite good adherence, with a drop-out rate of around 20% among patients, and the “easy to implement” method for lifestyle change in a real-world setting, with a five-meeting schedule in 12 months and without a personalized meal-planning. As for obesity counselling, we successfully managed NAFLD patients, with a “5 As” model consisting of the following: (i) asking important general information about patient's lifestyle and attitudes; (ii) assessing dietary and physical activity habits with validated tools in order to personalize and choose an adequate lifestyle change; (iii) advising patients about the importance of gradual behavior changes in everyday life in order to achieve a better health status; (iv) agreeing on realistic objectives about diet and exercise; and (v) assisting with regular follow-up management [27].

The 12-month duration of the intervention is one of the longest in this type of trial [9], which also takes into account of the difficulty to maintain patient compliance for all the 3-month periodical evaluations.

Our study has also some limitations that need to be addressed. The main limitation is the study design, which was not randomized. The allocation to the NAFLD patients group or to the healthy control group was based on the results of the baseline US liver examination. We have to acknowledge that US is not sensible enough to detect lower amounts of liver fat and that the possibility that some participants in the control group had some level of steatosis cannot be ruled out. Moreover, we acknowledge that we did not perform any additional noninvasive test, neither liver biopsy. According to most recent AASLD practice guidelines for the diagnosis management of NAFLD, controlled attenuation parameter (CAP) provides a good semiquantitative assessment of hepatic steatosis [28]; at the time of the study progress, no elastography instrument was available at the Department of Medicine, (removed for blind peer review). In order to calculate other noninvasive markers, such as NAFLD-fibrosis score or FIB-4 score, we needed platelet count, which was not performed among blood tests. We performed additional blood tests assessing mitochondrial respiratory parameters among a subgroup of patients [19] and of healthy subjects [18] according to approved study procedures. As detailed elsewhere, these tests had a good correlation with NAFLD presence and degree and with other clinical, anthropometric, and biochemical characteristics in the whole subgroup of the study patients and healthy subjects [29]. Since we aimed to assess the impact of a resource-saving intervention, suitable for a real-world setting, we decided to include NAFLD patients only in the intervention arm. On the other hand, the parallel observation of a control group of healthy subjects allowed us to argue that simple participation in a research study, with physical, instrumental, and laboratory assessments and general advice on healthy lifestyle, is not effective to lead the participants to modify their habits for preventing NAFLD occurrence. It is of note that the healthy controls had a fair mean Mediterranean dietary score at baseline, whereas NAFLD patients had not, but both groups had the same distribution of Medscore at the end of the study: the former group improved the score, whereas the latter did not.

Our lifestyle intervention was mainly oriented to dietary advice without personalised advice to participants on performing physical activity according to present guidelines.

To assess the impact of the intervention, we performed a per-protocol analysis instead of an intention to treat one, because of the lack of the outcome variables (liver steatosis, anthropometric, and laboratory measures and Medscore) in the subjects lost at follow-up. Although this approach may determine a selection bias, the relatively small number of lost subjects and the lack of significant differences between them and the subjects who completed the study at baseline evaluation, in both NAFLD patients and controls, allow us to be confident that no substantial bias altered substantially the results of the study. The number of patients who completed the study was large enough to detect a statistically significant effect of the lifestyle intervention, according to the a priori power analysis.

Finally, we used the US for the evaluation of the presence and severity of liver steatosis instead of a biopsy, which is the gold standard for diagnosis and follow-up of NAFLD/NASH [26]. However, liver biopsy is an invasive technique, which may determine harm to patients and therefore is not commonly used for lifestyle trials in subjects who are not suspected to have already developed NASH.

## 5. Conclusions

In conclusion, we conducted a Mediterranean-oriented lifestyle change intervention among NAFLD patients that showed a rather fair efficacy, good adherence, and feasibility in real-world settings. The improvement in liver steatosis in almost half of the patients was independent of changes in BMI or serum liver enzyme levels.

In future research, other professionals should be involved in the multidisciplinary team: a health professional with experience in the field of physical activity, to tailor the best physical training to each person and a psychologist to both improve our communications and overcome patients' barriers to lifestyle change. Still open questions are about the maintenance of lifestyle change in the long-term period and the reduction in NAFLD complications, which is the very last goal of such intervention strategies.

## Figures and Tables

**Table 1:** Characteristics of the NAFLD patients and healthy controls.

	NAFLD patients (*n* = 58)	Healthy controls (*n* = 73)	*P* ^*∗*^
Male/female, number	31/27	31/42	0.24
Age (years), mean ± SD	49.0 ± 7.9	46.5 ± 9.8	0.10
Education level, number (%)			
Primary school	22 (37.9)	17 (23.3)	
High school	24 (41.4)	26 (35.6)	0.01
University	9 (15.5)	28 (38.3)	
Not declared	3 (5.2)	2 (2.7)	
BMI (kg/m^2^), mean ± SD	31.4 ± 5.5	24.0 ± 3.8	<0.001
BMI category (BMI range), number (%)			
Underweight (<18.5)	0	5 (6.8)	<0.001
Normal weight (18.5–24.9)	3 (5.2)	39 (53.4)	
Overweight (25–29.9)	20 (34.5)	25 (34.2)	
Class I obesity (30–34.9)	26 (44.8)	4 (5.5)	
Class II obesity (35–39.9)	4 (6.9)	0	
Class III obesity (≥40)	5 (8.6)	0	
Waist circumference (cm), mean ± SD			
Males	107.3 ± 15.4	93.0 ± 9.1	<0.001
Females	101.9 ± 12.2	83.2 ± 9.9	<0.001
Smoking habit, number (%)			
No	37 (63.8)	50 (68.5)	0.47
Yes	9 (15.5)	6 (8.2)	
Quit	12 (20.7)	15 (20.5)	
Not declared	0	2 (2.7)	
Alcohol consumption			
No	14 (24.1)	17 (23.3)	0.40
Daily (≤2 alcoholic units)	7 (12.1)	15 (20.5)	
Occasionally	37 (63.8)	41 (56.2)	
Family history			
Cardiovascular diseases, number (%)			
No	25 (43.1)	41 (56.2)	0.12
Yes	32 (55.2)	31 (42.5)	
Not declared	1 (1.7)	1 (1.4)	
Metabolic syndrome			
No	47 (81.0)	59 (80.8)	0.81
Yes	10 (17.2)	11 (15.1)	
Not declared	1 (1.7)	3 (4.1)	
Diabetes			
No	48 (82.8)	64 (87.7)	0.45
Yes	9 (15.5)	8 (10.9)	
Not declared	1 (1.7)	1 (1.4)	
Cancer			
No	26 (44.8)	42 (57.5)	0.17
Yes	31 (53.4)	30 (41.1)	
Not declared	1 (1.7)	1 (1.4)	

^*∗*^Mann–Whitney and exact tests for continuous and categorical variables, respectively.

**Table 2:** Means (SD) of laboratory findings and dietary scores among NAFLD patients and healthy controls at baseline.

Parameters	NAFLD patients (*n* = 58)	Healthy controls (*n* = 73)	*P* ^*∗*^
Total cholesterol (mg/dL)	208 (34)	196 (39)	0.06
HDL	50 (12)	66 (18)	<0.001
LDL	128 (31)	114 (38)	0.02
Triglycerides (mg/dL)	152 (93)	82 (39)	<0.001
Glycemia (mg/dL)	98 (19)	85 (9)	<0.001
Insulinemia (*μ*U/mL)	15 (14)	5 (4)	<0.001
AST (U/L)	24 (12)	20 (9)	0.02
ALT (U/L)	42 (22)	29 (22)	<0.001
HOMA-IR index	3.8 (4.2)	1.1 (0.9)	<0.001
Medscore	12 (3)	15 (4)	<0.001

HDL: high density lipoprotein; LDL: low density lipoprotein; HOMA-IR: HOmeostatic Model Assessment for Insulin Resistance; Medscore: Mediterranean diet adherence score. ^*∗*^Mann–Whitney test.

**Table 3:** Anthropometric values, laboratory findings, and Medscore among NAFLD patients and healthy controls at baseline (T0) and at the end of the study (T12): means (SD) and ΔT0–T12 as means (SD) in the differences of the values at T0 and at T12.

	NAFLD patients (58)				Healthy controls (73)			
	T0	T12	ΔT0–T12	*P* ^*∗*^	T0	T12	ΔT0–T12	*P* ^*∗*^
BMI (kg/m^2^)	31.4 (5.5)	30.5 (5.6)	0.9 (1.2)^§^	<0.001	24.0 (3.8)	24.0 (3.8)	0.0 (0.9)^§^	0.97
Waist circumference (cm)	105 (14)	104 (13)	0.9 (4.7)^#^	0.16	87 (11)	88 (11)	−0.8 (3.8)^#^	0.04
Total cholesterol (mg/dL)	207 (34)	211 (35)	−3.6 (28)	0.29	196 (40)	200 (35)	−4.4 (24)	0.13
HDL	50 (12)	53 (14)	−3.2 (7.5)	<0.01	66 (18)	66 (17)	−0.4 (8.1)	0.43
LDL	128 (31)	132 (29)	−3.7 (23)	0.22	114 (38)	117 (34)	−3.1 (22)	0.17
Triglycerides (mg/dL)	152 (96)	128 (58)	24 (76)	0.06	82 (39)	86 (43)	−4.1 (31)	0.60
Glycemia (mg/dL)	98 (19)	99 (20)	−0.5 (14)	0.57	85 (9)	85 (10)	0.2	0.47
Insulinemia (*μ*U/mL)	16 (15)	13 (9)	2.4 (11)	0.12	5 (4)	5 (4)	−0.1	0.94
AST (U/L)	22 (10)	22 (8)	0.2	0.71	19 (7)	21 (8)	−1.4 (7)	0.05
ALT (U/L)	41 (21)	40 (21)	1.6 (21)	0.43	29 (19)	30 (19)	−0.7 (15)	0.35
HOMA-IR index	3.9 (4.5)	3.6 (3.2)	0.3 (4.2)	0.23	1.1 (0.9)	1.1 (0.9)	0.0	0.86
Medscore	12 (3)	16 (3)	−3.2 (3.1)^§^	<0.001	15 (4)	15 (4)	−0.2^§^	0.20

^§^ *P* < 0.001 and ^#^*P* < 0.01 for the comparison between the differences among NAFLD patients and healthy controls (according to Wilcoxon nonparametric test for paired data). ^*∗*^Mann–Whitney test.

**Table 4:** Anthropometric values, laboratory findings, and scores at baseline (T0) and end of the intervention (T12) among NAFLD patients with regression of steatosis and in those with stable or worsening steatosis, according to liver US evaluation: means (SD) and ΔT0–T12 as means (SD) in the differences between the values at T0 and at T12.

	NAFLD patients with regression of steatosis (*n* = 26)				NAFLD patients with stability or worsening of steatosis (*n* = 32)			
	T0	T12	ΔT0–T12	*P* ^*∗*^	T0	T12	ΔT0–T12	*P* ^*∗*^
BMI (kg/m^2^)	31 (7)	30 (7)	1.0 (1)	<0.001	32 (4)	31 (4)	0.8 (1)	<0.001
Waist circumference (cm)	104 (19)	103 (16)	1.2 (6)	0.17	105 (10)	105 (10)	0.6 (4)	0.57
Total cholesterol (mg/dL)	204 (25)	214 (37)	−9.6 (29)	0.12	210 (41)	209 (34)	1.6 (27)	0.95
HDL	54 (13)	58 (17)	−4.4 (9)	0.03	47 (10)	49 (10)	−2.2 (6)	0.10
LDL	124 (26)	130 (30)	−5.7 (23)	0.20	131 (35)	133 (29)	−2.1 (24)	0.60
Triglycerides (mg/dL)	141 (110)	121 (62)	20.1 (84)	0.56	162 (82)	133 (54)	28.2 (69)	0.04
Glycemia (mg/dL)	101 (23)	102 (26)	−1.3 (19)	0.40	96 (14)	96 (13)	0.1 (7)	0.93
Insulinemia (*μ*U/mL)	10 (6)	10 (7)	0.5 (5)	0.22	21 (18)	16 (9)	4.0 (14)	0.32
AST (U/L)	21 (7)	21 (7)	0.0 (6)	0.87	23 (12)	23 (8)	0.4 (9)	0.68
ALT (U/L)	38 (18)	39 (21)	−1.2 (15)	0.75	45 (23)	41 (21)	4.0 (15)	0.14
HOMA-IR index	2.7 (2.6)	3.4 (4.1)	−0.7 (4.1)	0.48	5.0 (5.4)	3.8 (2.2)	1.2 (4.2)	0.35
Medscore	13 (4)	16 (4)	−3.3 (3.1)	<0.001	12 (3)	15 (3)	−3.1 (3.0)	<0.001

No statistically significant value for the comparison between ΔT0 and T12 among NAFLD patients with regression of steatosis vs. NAFLD patients with stability or worsening of steatosis (according to Wilcoxon nonparametric test for paired data). ^*∗*^Mann–Whitney test.

## Data Availability

The data used to support the findings of the study are available from the corresponding author upon request.

## Supplementary Materials

Supplementary Materials Table S1: criteria for score assignment in Medscore, according to selected food frequency consumption. Ethical approval (for reviewers only): PDF of the approval by the local Ethical Committee of Brescia District, on 26^th^ January 2017, for the conduction of the study. Supplementary File 1 (for reviewers only): copy of the booklet provided to each NAFLD patient. Supplementary File 2 (for reviewers only): copy of the booklet provided to each healthy control.
