# Supplementary material for: Mediterranean-Oriented Dietary Intervention Is Effective to Reduce Liver Steatosis in Patients with Nonalcoholic Fatty Liver Disease: Results from an Italian Clinical Trial
Source: Int J Clin Pract. 2024 Jan 25;2024:8861126. doi: 10.1155/2024/8861126 (PMC10834092; doi:10.1155/2024/8861126)

CONTATTI:

Segreteria Gastroenterologia Spedali Civili
Tel : 030 399.5241
E-mail: gastroenterologia@asst-spedalicivili.it

La riproduzione totale o parziale di questa pubblicazione è subordinata alla autorizzazione delle Dottoresse:
BARBARA ZANINI
MONYA MARULLO

Steatosi Epatica non alcolica e da epatite C: Epidemiologia nutrizionale e Lifestyle mediciNe

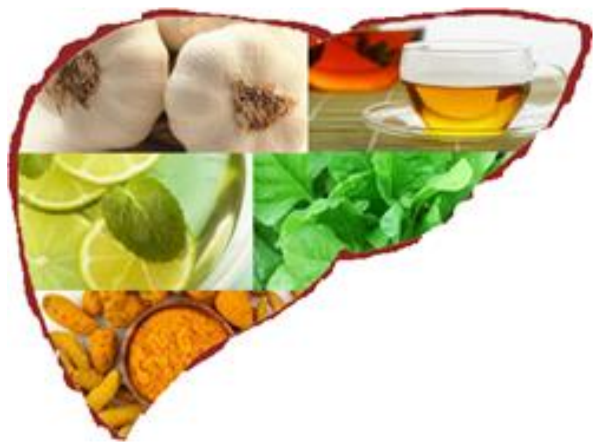

GUIDA PER IL PAZIENTE

- Febbraio 2017 -

COGNOME________________________________

NOME____________________________________

DATA NASCITA____________________________

STEATOSI EPATICA

Definizione: La steatosi epatica, conosciuta dalla popolazione generale con il termine "fegato grasso", è una condizione patologica in cui aumenta la percentuale di grasso a livello epatico >10% che predispone il soggetto al rischio di sviluppare serie complicanze: cirrosi e carcinoma epato-cellulare.

Epidemiologia:
La steatosi epatica è una condizione presente in circa il 55% dei soggetti affetti da HCV (virus della epatite C), e nel 25-45% della popolazione che presenta una condizione di sovrappeso e/o obesità con un aumento della adiposità viscerale. In questi ultimi soggetti si parla di "Steatosi epatica non alcolica" *(NAFLD-**N**on **A**lcohlic **F**atty **L**iver **D**isease).*

Terapia:
La steatosi epatica aumenta il rischio di sviluppare nel tempo diabete e/o malattie cardiovascolari.
Ad oggi non esistono terapie farmacologiche approvate per la cura della steatosi epatica di origine non alcolica, quindi, secondo la OMS, è necessario pianificare ed attuare interventi mirati sugli scorretti stili di vita, in particolare diete sbagliate e sedentarietà.

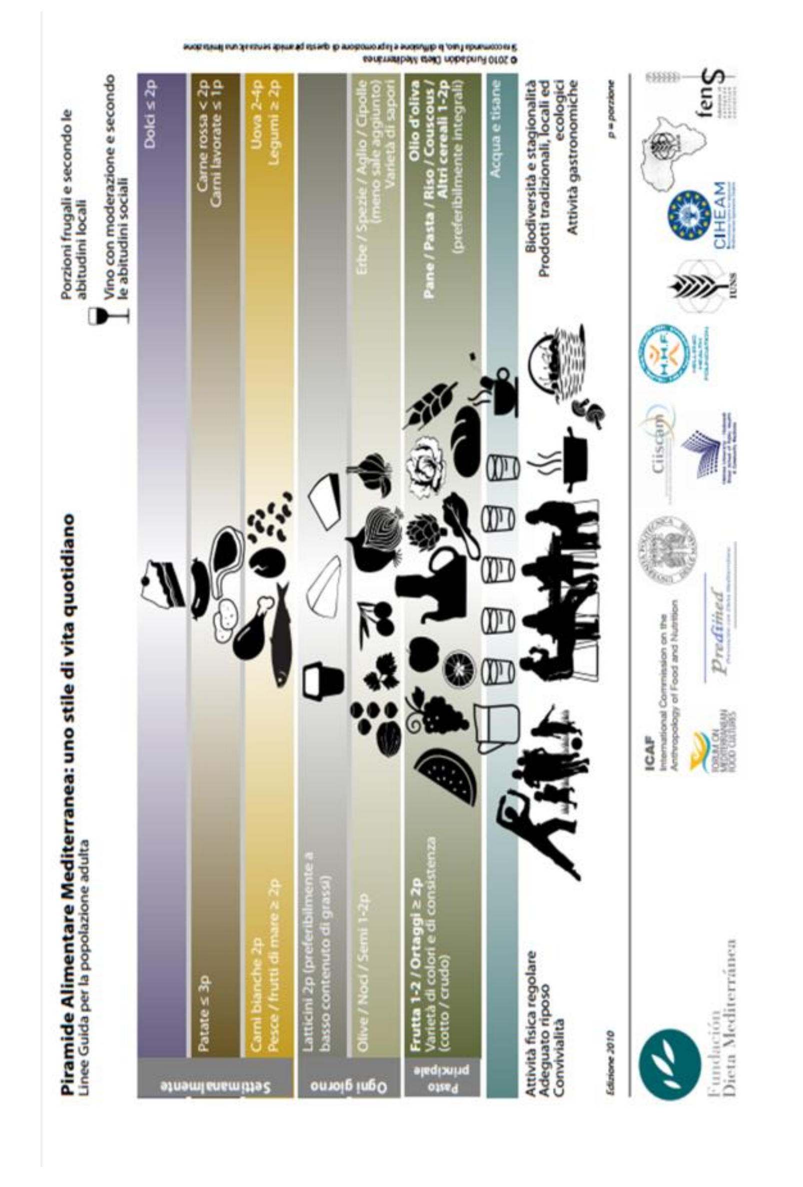

LATTE E LATTICINI

Il latte e i suoi derivati sono considerati alimenti cardine della dieta mediterranea e per questa ragione la loro assunzione è fortemente consigliata, nell'ambito di una alimentazione bilanciata, per tutte le fasce di età. La piramide alimentare della dieta mediterranea moderna elaborata dal CIISCAM (Centro Interuniversitario Internazionale di Studi sulle Culture Alimentari Mediterranee), consiglia l'assunzione di 2 porzioni giornaliere (preferibilmente a ridotto contenuto di grassi).

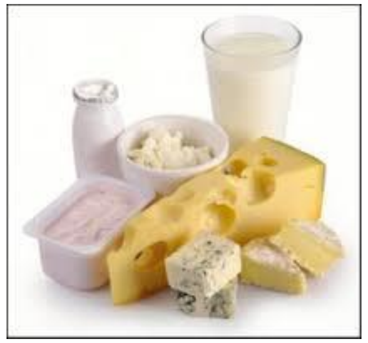

ALCOL

Per una buona prevenzione oncologica la raccomandazione sarebbe di evitare il consumo di bevande alcoliche. Tuttavia, se si vogliono consumare bevande alcoliche è bene consumarne piccole quantità solo occasionalmente. Le quantità indicate da non superare sono 1 unità alcolica al giorno per le donne e 2 unità al giorno per gli uomini.

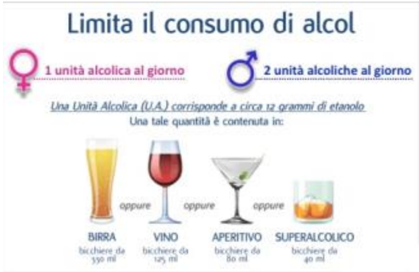

Porzioni *"Dieta Mediterranea"*:

Porzioni frugali e secondo le abitudini locali

Vino con moderazione.

MALATTIE CRONICHE NON TRASMISSIBILI (MCNT)

Le malattie croniche non trasmissibili costituiscono la principale causa di morte nel mondo. Dei 57 milioni di decessi registrati a livello mondiale nel 2008, 36 milioni (63%) sono stati causati da queste malattie:

1. **DIABETE**
2. **MALATTIE CARDIOVASCOLARI**
3. **MALATTIE RESPIRATORIE CRONICHE**
4. **TUMORI**

L'Organizzazione Mondiale della Sanità ha elaborato un "Piano di Azione Globale" per contrastare queste malattie ed intervenire in particolare con strategie mirate sugli:

STILI DI VITA SCORRETTI

A. **TABACCO**– 6 milioni di persone muoiono ogni anno a causa del tabacco, sia per il consumo attivo che passivo

B. **CONSUMO DANNOSO DI ALCOL–** Muoiono circa 2,3 milioni di persone l'anno. Più della metà di queste morti è dovuta al cancro, malattie cardiovascolari e cirrosi epatica.

C. **INATTIVITA' FISICA**– muoiono circa 3,2 milioni di persone ogni anno a causa di un incremento del rischio di mortalità (tra il 20% e 30%) per tutte le cause.

D. **ERRATE ABITUDINI ALIMENTARI–** L'eccessivo consumo di alimenti ad alta densità calorica (in particolare junk-food *"cibo spazzatura")* sono tra le cause maggiori di sovrappeso e obesità.

THE MEDITERRANEAN DIET SCORE

PRIMA VISITA	
OBIETTIVO N.1	
OBIETTIVO N.2	
OBIETTIVO N.3	

FOLLOW-UP 3 MESI	
OBIETTIVO N.1	
OBIETTIVO N.2	
OBIETTIVO N.3	

FOLLOW-UP 6 MESI	
OBIETTIVO N.1	
OBIETTIVO N.2	
OBIETTIVO N.3	

FOLLOW-UP 9 MESI	
OBIETTIVO N.1	
OBIETTIVO N.2	
OBIETTIVO N.3	

OLI E CONDIMENTI

Scegli la qualità e limita la quantità. Per stare bene è necessario introdurre con l'alimentazione una certa quantità di grassi, ma è altrettanto opportuno non eccedere. I grassi, oltre a fornire energia apportano acidi grassi essenziali della famiglia omega-6 (acido linoleico) e della famiglia omega-3 (acido linolenico) e favoriscono l'assorbimento delle vitamine liposolubili A, D, E, K e dei carotenoidi.

Porzioni *"Dieta Mediterranea":*

Da consumare nel pasto principale (olio di oliva)

ALLA BASE DELLA PIRAMIDE ALIMETARE

Adeguato riposo

Convivialità

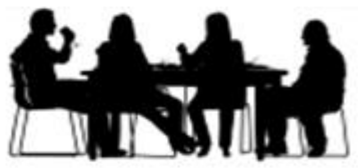

Attività fisica regolare

Adulti (18-64 anni) almeno 150 minuti a settimana attività moderata oppure 75 minuti di attività vigorosa (o condizioni equivalenti delle 2) in sessioni di almeno 10 minuti per volta con rafforzamento dei maggiori gruppi muscolari da svolgere almeno 2 volte a settimana

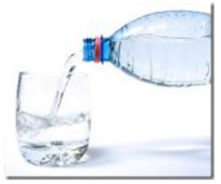

Acqua e tisane

CARNI BIANCHE

Porzioni "*Dieta Mediterranea*":

≥ 2 porzioni a settimana

UOVA

Porzioni "*Dieta Mediterranea*":

2-4 porzioni a settimana

CARNE ROSSA E CARNI LAVORATE

Limita la carne rossa e evita la carne lavorata. Questi sono gli obiettivi della sanità pubblica. Secondo le raccomandazioni sulla prevenzione del cancro la WCRF le carni rosse non deve superare i 300g alla settimana, che corrispondono a circa due porzioni settimanali. Per "*carne lavorata*" si intende la carne conservata dal fumo, la polimerizzazione o la salatura o l'aggiunta di conservanti chimici, inclusa quella contenuta negli alimenti trasformati.

World Cancer Research Fund International

Porzioni "*Dieta Mediterranea*":

‹ 2 porzioni a settimana (carne rossa)

≤ 2 porzioni a settimana (carni lavorate)

ERBE AROMATICHE E SPEZIE

Utile sarebbe utilizzarle nella preparazione delle diverse pietanze in sostituzione del sale. Ottime per insaporire.

DOLCI

Porzioni "*Dieta Mediterranea*":

≤ 2 porzioni a settimana

ATTIVITA' FISICA QUOTIDIANA (IPAQ)

TOTALE MET	

PRIMA VISITA	
OBIETTIVO N.1	

FOLLOW-UP 3 MESI	
OBIETTIVO N.1	

FOLLOW-UP 6 MESI	
OBIETTIVO N.1	

FOLLOW-UP 9 MESI	
OBIETTIVO N.1	

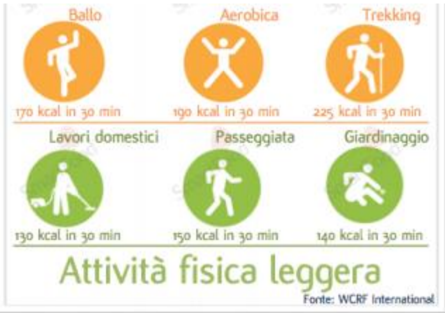

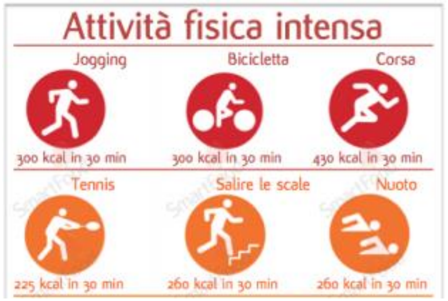

DIETA MEDITERRANEA

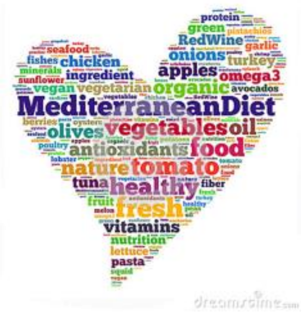

patrimonio culturale
immateriale dell'Umanità
(UNESCO 2010)

LA STORIA:

Anni '30
Lorenzo Piroddi:
connessione tra alimentazione e diabete/obesità
Anni '50
Ancel Keys:
popolazione del Sud Italia più sana dei cittadini di NY
•Più di 50 studi confermano "potere" della DM (Dieta Mediterranea)

I PRINCIPI CARDINE:

1. Mangiare prevalentemente cibi di origine vegetale: verdura, legumi e frutta fresca e secca, cereali integrali
2. Impiego massiccio di olio di oliva, a fronte di modesta assunzione di grassi saturi
3. Discreta assunzione di pesce e pollame (2-3 volte/sett)
4. Limitato consumo di carni rosse (poche volte al mese) e modica assunzione di prodotti caseari (yogurt e formaggi)
5. Uso di spezie e di erbe aromatiche invece del sale
6. (opzionale) bere vino rosso a pasto in piccole quantità -CONSULTARE IL MEDICO
7. Condividi il cibo con famiglia e amici

PESCE-FRUTTI DI MARE

I prodotti della pesca comunemente consumati si suddividono in:

- ***Pesci***
- ***Crostacei***
- ***Molluschi***
- ***Echinodermi***

Il **pesce azzurro** *(Aguglia, Alaccia, Alice o acciuga, Aringa, Costardella, Cheppia Ricciola, Cicerello, Lanzardo, Sardina, Bianchetti, Sgombro, Papalina, Sauro, Tonno, Palamita, Pesce spada, Tonno rosso)* è una varietà ittica con determinate caratteristiche fisiche (squame colorate di blu sul dorso e argentee sulla pancia) nonché nutrizionali. La carne del pesce azzurro è molto facile da digerire nonché ricca di grassi buoni, ovvero gli omega3, contiene inoltre: selenio, calcio, iodio, fosforo, potassio, selenio, fluoro, zinco, vitamine A e B. Gli omega3 sono grassi buoni che favoriscono i livelli di diminuzione del colesterolo, favoriscono la pulizia delle arterie, prevengono i tumori al colon ed al pancreas, diminuiscono le probabilità di soffrire di demenza senile ed inoltre combattono l'invecchiamento precoce.

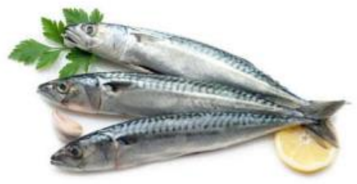

Porzioni "*Dieta Mediterranea*":
≥ 2 porzioni a settimana

LEGUMI

Con lo slogan *"semi nutrienti per un futuro sostenibile"*, l'Assemblea Generale delle Nazioni Unite ha dichiarato il ***2016 l'anno Internazionale dei Legumi.***

I legumi, oltre ad essere ricchi di proteine, il doppio di quelle presenti nel grano e il triplo di quelle del riso, sono un'importante fonte di micronutrienti, aminoacidi e vitamine del gruppo B. Sono alimenti a basso contenuto di grassi e ricchi di sostanze nutritive e di fibra solubile. Ricchi di ferro e zinco vengono considerati alimenti importanti per la lotta contro l'anemia di donne e bambini. Il consiglio di consumo è di almeno 3 volte alla settimana.

Porzioni "*Dieta Mediterranea*":

≥ 2 porzioni a settimana

ELENCO LEGUMI

Ceci	Fagioli	Soia	Lupini
Piselli	Lenticchie	Fave	Taccole

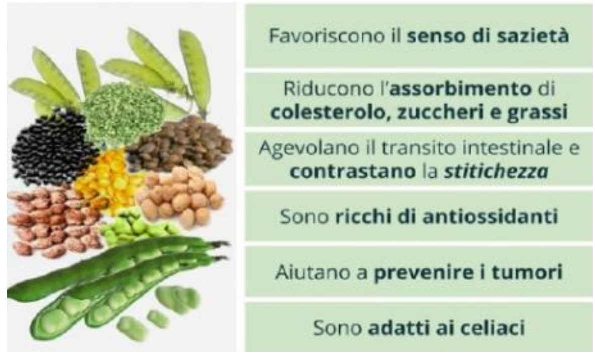

H.E.P.
Healthy **E**ating **P**late

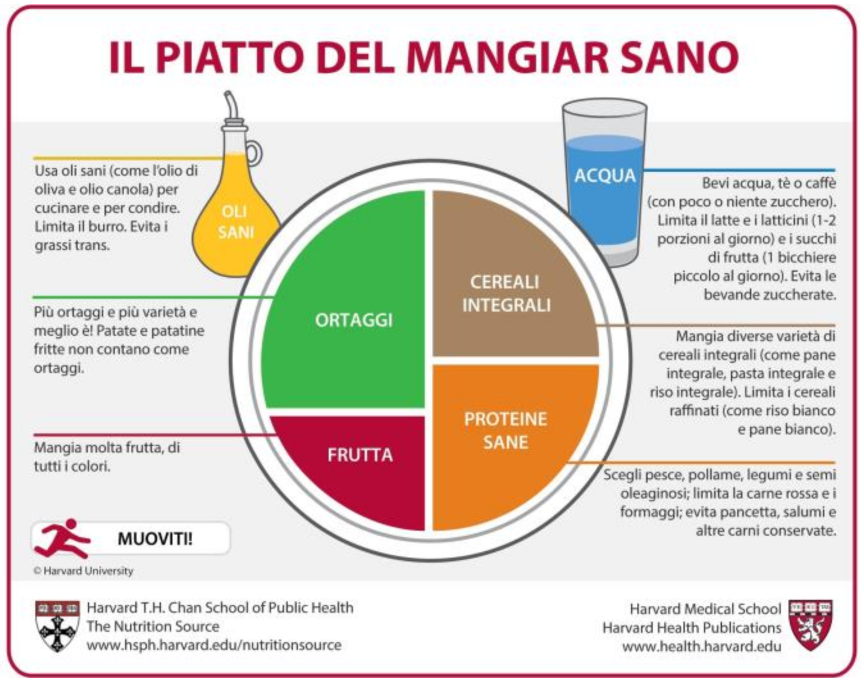

E' dimostrato che seguire una dieta mediterranea è associato a:

- -Riduzione di mortalità (tutte le cause)
- -Riduzione di mortalità cardiovascolare
- -Riduzione di mortalità correlata a tumori
- -Riduzione di tumore (incidenza e recidive)
- -Riduzione di incidenza di Morbo di Parkinson e Alzheimer
- -Riduzione casi di diabete, ipertensione e obesità

CEREALI INTEGRALI (loro derivati, tuberi compresi)

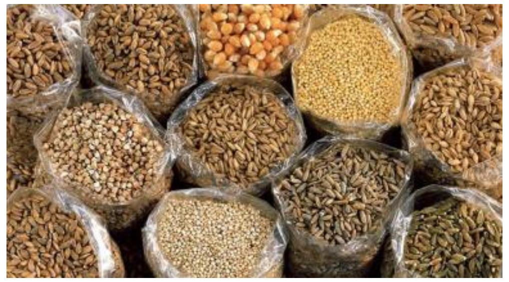

Per cereali integrali si intendono tutti i cereali o pseudo-cereali che in natura si trovano sotto forma di chicco e che vengono consumati senza subire processi di abburattamento (raffinazione) da parte dell'industria alimentare. Questo processo impoverisce il cereale di importanti nutrienti, in particolare Vitamine B_1 B_2 B_6, PP , Sali minerali e fibra.

Gradi di raffinazione

FARINA	integrale	2	1	0	00

Come riconosco un cereale realmente integrale?
Leggi l'etichetta! Lista degli ingredienti.

VERO INTEGRALE

Ingredienti:
Farina integrale di grano tenero macinata a pietra 81,6%, pasta madre di farina integrale di grano tenero 13% (farina integrale di grano tenero, acqua), olio extravergine di oliva 2,1%, lievito (Saccharomyces cerevisiae), sale marino, malto d orzo.

Porzioni *"Dieta Mediterranea"*:
Pane, pasta, riso, couscous, altri cereali preferibilmente integrali:

- 1-2 porzioni ai pasti principali
- ≤ 3 porzioni alla settimana (patate)

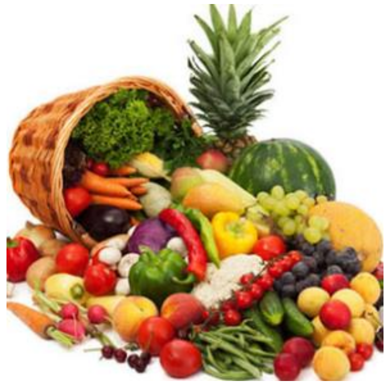

FRUTTA, VERDURA E ORTAGGI.

I prodotti ortofrutticoli oltre ad essere una importante fonte di vitamine e sali minerali, contengono delle molecole definite bioattive, che si pensa possano esercitare azione protettiva, prevalentemente di tipo antiossidante. La frutta apporta sostanzialmente pro-vitamina A e vitamina C, gli ortaggi apportano significative quantità di pro-vitamina A, vitamina C e acido folico.

PORZIONI GIORNALIERE (L.A.R.N.)
Frutta 1-2 porzioni pasti principali
Ortaggi/verdura ≥2 porzioni pasti principali

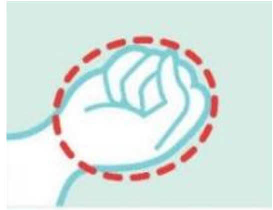

Insalate a foglia: 80 g.
Verdure crude o cotte: 200 g.
Ortaggi crudi o cotti: 200 g.

Frutta fresca: 150g.
Frutta secca e/o zuccherina: 30g.

Porzione visiva:
Frutta fresca (1 pugno)
Verdura e/o ortaggi a cotto (1 pugno)
Verdura in foglia a crudo (2 pugni)

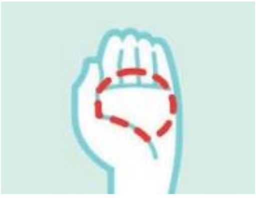

Supplement: Supplementary Materials — Table S1: criteria for score assignment in Medscore, according to selected food frequency consumption. Ethical approval (for reviewers only): PDF of the approval by the local Ethical Committee of Brescia District, on 26th January 2017, for the conduction of the study. Supplementary File 1 (for reviewers only): copy of the booklet provided to each NAFLD patient. Supplementary File 2 (for reviewers only): copy of the booklet provided to each healthy control. [file 8861126.f1.zip › Supplementary file 1.pdf]
