# Supplementary material for: Mediterranean-Oriented Dietary Intervention Is Effective to Reduce Liver Steatosis in Patients with Nonalcoholic Fatty Liver Disease: Results from an Italian Clinical Trial
Source: Int J Clin Pract. 2024 Jan 25;2024:8861126. doi: 10.1155/2024/8861126 (PMC10834092; doi:10.1155/2024/8861126)
Supplement: Supplementary Materials — Table S1: criteria for score assignment in Medscore, according to selected food frequency consumption. Ethical approval (for reviewers only): PDF of the approval by the local Ethical Committee of Brescia District, on 26th January 2017, for the conduction of the study. Supplementary File 1 (for reviewers only): copy of the booklet provided to each NAFLD patient. Supplementary File 2 (for reviewers only): copy of the booklet provided to each healthy control. [file 8861126.f1.zip › Supplementary file 2.pdf]

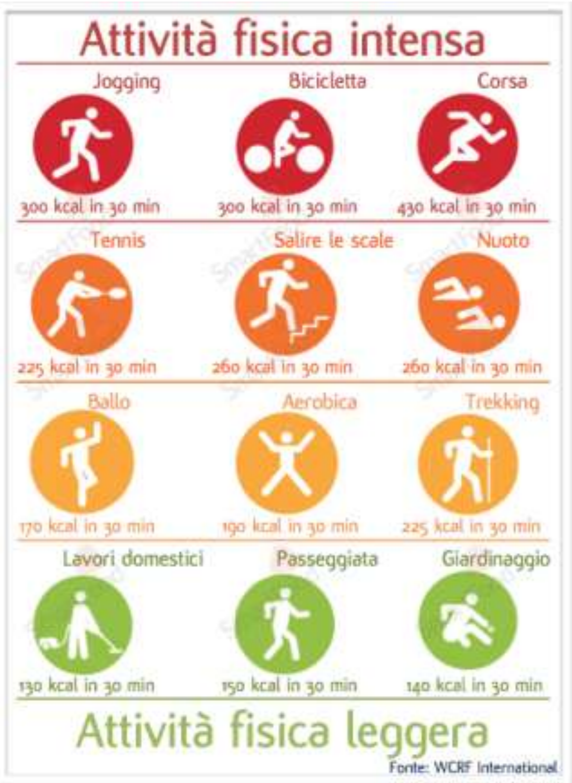

Adulti (18-64 anni) almeno 150 minuti a settimana attività moderata oppure 75 minuti di attività vigorosa (o condizioni equivalenti delle 2) in sessioni di almeno 10 minuti per volta con rafforzamento dei maggiori gruppi muscolari da svolgere almeno 2 volte a settimana

CONTATTI:

Segreteria Gastroenterologia Spedali Civili
Tel : 030 399.5241
E-mail: gastroenterologia@asst-spedalicivili.it

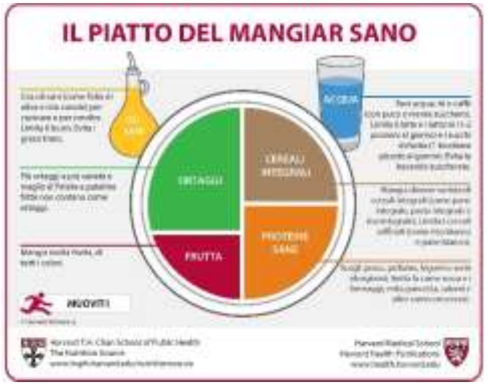

La riproduzione totale o parziale di questa pubblicazione è subordinata alla autorizzazione delle Dottoresse:

BARBARA ZANINI
MONYA MARULLO

Steatosi Epatica non alcolica e da epatite C: Epidemiologia nutrizionale e Lifestyle mediciNe

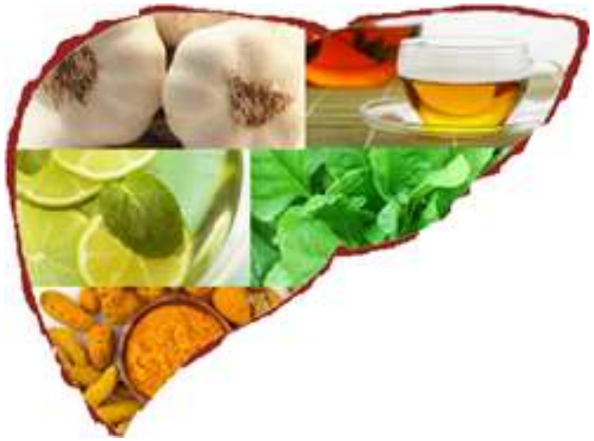

Guida per il paziente

Piramide Alimentare Mediterranea: uno stile di vita quotidiano

Linee Guida per la popolazione adulta

Porzioni frugali e secondo le abitudini locali

Vino con moderazione e secondo le abitudini sociali

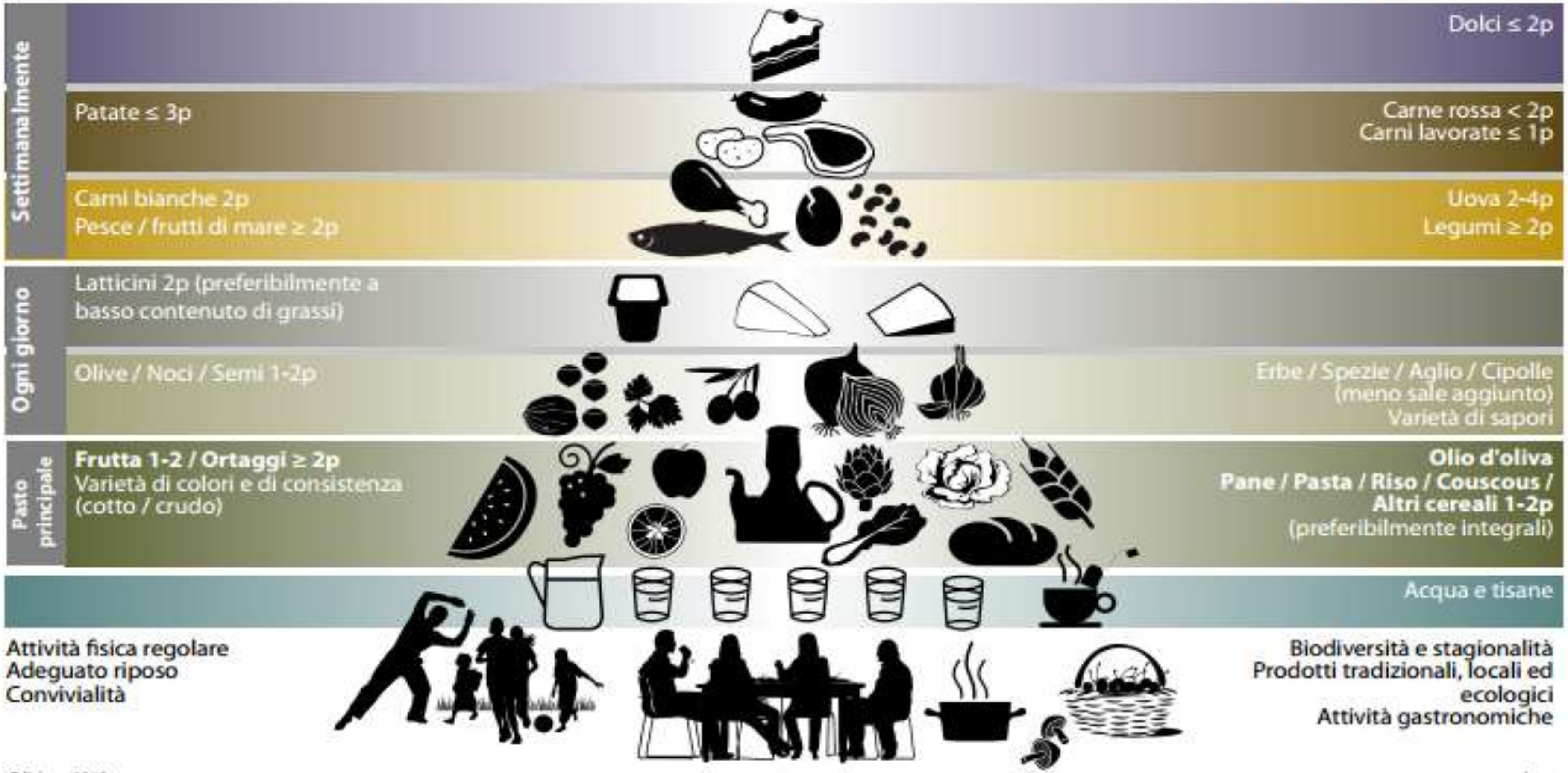

Attività fisica regolare
Adeguato riposo
Convivialità

Biodiversità e stagionalità
Prodotti tradizionali, locali ed ecologici
Attività gastronomiche

Edizione 2010

p = porzione

© 2010 Fundación Dieta Mediterránea
Si raccomanda l'uso, la diffusione e la promozione di questa piramide senza alcuna limitazione

Fundación Dieta Mediterránea

ICAF International Commission on the Anthropology of Food and Nutrition

Forum on Mediterranean Food Cultures

Predimed

Ciiscam

H.H.F. Hellenic Health Foundation

IUNS

CIHEAM

fens
