# Supplementary material for: Mediterranean-Oriented Dietary Intervention Is Effective to Reduce Liver Steatosis in Patients with Nonalcoholic Fatty Liver Disease: Results from an Italian Clinical Trial
Source: Int J Clin Pract. 2024 Jan 25;2024:8861126. doi: 10.1155/2024/8861126 (PMC10834092; doi:10.1155/2024/8861126)
Supplement: Supplementary Materials — Table S1: criteria for score assignment in Medscore, according to selected food frequency consumption. Ethical approval (for reviewers only): PDF of the approval by the local Ethical Committee of Brescia District, on 26th January 2017, for the conduction of the study. Supplementary File 1 (for reviewers only): copy of the booklet provided to each NAFLD patient. Supplementary File 2 (for reviewers only): copy of the booklet provided to each healthy control. [file 8861126.f1.zip › Table S1 IJCP REV 1.docx]

| **Food** | **Assigned score** | | |
| --- | --- | --- | --- |
|  | **0** | **1** | **2** |
| Whole pasta | Less than 50% of the pasta consumption | More than 50% of the pasta consumption | / |
| Whole bread | <3 times /week | ≥ 3 times/week | / |
| Vegetables | <6 times/week | 6 times/week | >6 times/week |
| Fruits | <5 times/week | 5 times/week | >5 times/week |
| Pulses | <1 time/week | 1 time/week | >1 time/week |
| Fish | <1 time/week | ≥ 1 time/week | / |
| Nuts | <1 time/week | ≥1 and <7 times/week | ≥7 times/week |
| Olive oil | <7 times/week | 7 times/week | >7 times/week |
| Wine | >7 times/week | ≤7 times/week | / |
| Processed meat | >1 time/week | ≤1 time/week | / |
| Red meat | >1 time/week | ≤1 time/week | / |
| Poultry and eggs | ≥5 times/week | <5 times /week |  |
| Sugary drinks | occasionally or more | never | / |
| Confectionery | >1 time/week | ≤1 time/week | / |
| Cheese | >3 times/week | ≤3 times/week | / |
| Yogurt | ≤1 or >7 times/week | >1 and ≤ 7 times/week | / |
| Butter | ≥ 3 times/week | <3 times /week | / |
| Spirits | >1 time/week | ≤1 time/week | / |
| Water | <3 glasses/day | ≥3 and <6 glasses/day | ≥6 glasses/day |
|  |  |  |  |

Table S1. Criteria for score assignment in Medscore, according to selected food frequency consumption
